# Differential Regulation of an OsIspH1, the Functional 4-Hydroxy-3-Methylbut-2-Enyl Diphosphate Reductase, for Photosynthetic Pigment Biosynthesis in Rice Leaves and Seeds

**DOI:** 10.3389/fpls.2022.861036

**Published:** 2022-04-13

**Authors:** Yeo Jin Lee, Jae Kwang Kim, Seung-A Baek, Ji-Su Yu, Min Kyoung You, Sun-Hwa Ha

**Affiliations:** ^1^Department of Genetics and Biotechnology, Graduate School of Biotechnology, College of Life Sciences, Kyung Hee University, Yongin, South Korea; ^2^Division of Life Sciences, Bio-Resource and Environmental Center, Incheon National University, Incheon, South Korea

**Keywords:** carotenoids and chlorophylls, duplicated pseudogene, functional complementation, methylerythritol 4-phosphate pathway, OsIspH1, OsIspH2, rice

## Abstract

The methylerythritol 4-phosphate (MEP) pathway is responsible for providing common precursors for the biosynthesis of diverse plastidial terpenoids, including chlorophylls, carotenoids, and phytohormones, in plants. In rice (*Oryza sativa*), the last-step genes encoding 4-hydroxy-3-methylbut-2-enyl diphosphate reductase [HDR/isoprenoid synthesis H (IspH)] have been annotated in two genes (*OsIspH1* and *OsIspH2*) in the rice genome. The spatial transcript levels indicated that *OsIspH1* is highly expressed in all tissues at different developmental stages, whereas *OsIspH2* is barely expressed due to an early stop in exon 1 caused by splicing error. OsIspH1 localized into plastids and *osisph1*, a T-DNA inserted knockout mutant, showed an albino phenotype, indicating that *OsIspH1* is the only functional gene. To elucidate the role of *OsIspH1* in the MEP pathway, we created two single (H145P and K407R) and double (H145P/K407R) mutations and performed complementation tests in two *hdr* mutants, including *Escherichia coli* DLYT1 strains and *osisph1* rice plants. The results showed that every single mutation retained HDR function, but a double mutation lost it, proposing that the complementary relations of two residues might be important for enzyme activity but not each residue. When overexpressed in rice plants, the double-mutated gene, *OsIspH1^MUT^*, reduced chlorophyll and carotenoid biosynthesis in the leaves and seeds. It confirmed the crucial role of *OsIspH1* in plastidic terpenoid biosynthesis, revealing organ-specific differential regulation of *OsIspH1* in rice plants.

## Introduction

Plants synthesize an enormous variety of terpenoids that serve as growth regulators (cytokines, gibberellins, abscisic acids, strigolactones, and brassinosteroids), photosynthetic and respiratory components (chlorophylls, carotenoids, prenylquinones, and ubiquinone), membranous sterols, and other secondary metabolites (Nagegowda and Gupta, 2020). All terpenoids are derived by the condensation of two universal precursors, namely, isopentenyl diphosphate (IPP, C5) and dimethylallyl diphosphate (DMAPP, C5). While most organisms retain one pathway for the biosynthesis of these isoprene building units, plants have two independent pathways, namely, the cytosolic mevalonate (MVA) and plastidic methylerythritol 4-phosphate (MEP) pathways (Hemmerlin et al., 2012). The latter has been evolutionally acquired due to endosymbiosis with prokaryotes and consists of seven enzymatic steps that start with the condensation of pyruvate (C3) and glyceraldehyde 3-phosphate (C3) derived from glycolysis into 1-deoxy-D-xylulose 5-phosphate (DXP, C5) by the action of DXP synthase (DXS) and end with the production of IPP and DMAPP from 4-hydroxy-3-methylbut-2-enyl diphosphate (HMBPP, C5) by the catalyst of HMBPP reductase (HDR), also known as isoprenoid synthesis H (IspH) or lysis-tolerant B (LytB; Vranova et al., 2013).

The MEP genes have been reported mainly in photosynthetic organisms, such as cyanobacteria, algae, and plants (Hemmerlin et al., 2012). However, Apicomplexa (malaria causative *Plasmodium* spp.), a non-photosynthetic protist, possesses MEP genes, although its vestigial chloroplasts and apicoplasts result from convergent reduction (Mathur et al., 2021). Bacterial MEP genes have also been discovered in *Eubacteria*, such as *Escherichia coli* and *Streptomyces* species, *Chlamydia* (obligate intracellular bacteria causing a sexually transmitted infection), and *Mycobacterium tuberculosis* (tuberculosis causative bacteria) (Rohmer et al., 1993; Grieshaber et al., 2004; Eoh et al., 2009). Therefore, MEP genes, including *IspH*, have been identified as promising targets for developing algicides, herbicides, anti-malaria drugs, and bactericides to combat infectious diseases, such as *Chlamydia* and tuberculosis (Singh et al., 2007; Masini and Hirsch, 2014; Wang and Dowd, 2018).

At present, several *IspH* genes have been characterized in diverse species, including cyanobacteria (*Synechocystis* strain PCC 6803, Cunningham et al., 2000), *E. coli* (Altincicek et al., 2002), *Aquifex* (*Aquifex aeolicus*, Wang et al., 2010), green microalgae (*Botryococcus braunii*, Uchida et al., 2018), ginkgo (*Ginkgo biloba*, Kim et al., 2008), red pine (*Pinus densiflora*, Kim et al., 2009), tobacco (*Nicotiana benthamiana*, Page et al., 2004), tomato (*Lycopersicon esculentum*, Botella-Pavia et al., 2004), *Arabidopsis* (*Arabidopsis thaliana*, Hsieh and Goodman, 2005), corn (*Zea mays*, Lu et al., 2012), danshen (*Salvia miltiorrhiza*, Hao et al., 2013), melon (*Cucumis melo*, Saladie et al., 2014), sweet wormwood (*Artemisia annua*, Ma et al., 2017), and rice (*Oryza sativa*, Liu et al., 2020). Among them, the protein structures were first examined in bacterial systems, such as *A. aeolicus* and *E. coli*, to discover *IspH* inhibitors that have potential for novel cancer immunotherapy because the substrate of *IspH*, HMBPP, has been known as a potent phosphoantigen that activates γδ T cells to kill tumor cells (Hintz et al., 2001; Grawert et al., 2004; Rao and Oldfield, 2016). IspH is identified as a 4Fe-4S cluster-containing protein with a trefoil-like structure, and its key residues are elucidated: the Fe-S cluster acts as an electron transfer cofactor in the active site, with direct involvement of the fourth Fe in substrate binding and catalysis, and it requires coordination with three Cys-residues for ligand binding (Wang et al., 2010; Hsieh et al., 2014; Rao and Oldfield, 2016).

*In planta* IspH functions have been reported for diverse terpenoid production in several plants. The overexpression of the *L. esculentum* HDR gene (*LeHDR*) increased carotenoids in tomato fruits during ripening and *Arabidopsis* seedlings during de-etiolation, and co-expression with taxadiene synthase gene increased taxadiene levels, indicating synergistic effects (Botella-Pavia et al., 2004). The overexpression of the *S. miltiorrhiza* HDR gene (*SmHDR1*) enhanced tanshinone production in cultured hairy roots of *S. miltiorrhiza* Bge. F. alba (Hao et al., 2013). The overexpression of the *A. annua* HDR gene (*AaHDR1*) increased the contents of artemisinin, arteannuin B, other sesquiterpenes, and monoterpenes, while its antisense-induced suppression exhibited opposite effects (Ma et al., 2017). Recently, the overexpression of the *G. biloba* HDR2 gene (*GbHDR2/GbIspH2*) in *Nicotiana tabacum* cv. Xanthi resulted in an increased photosynthetic rate through the upregulation of biosynthetic genes for photosynthetic pigments, including chlorophylls and carotenoids, as well as the elevated contents of duvatrienediol, the major diterpene of the *N. tabacum* leaf surface (Kim et al., 2021). However, the loss-of-function studies on *IspH* genes have been reported in several plants. Tobacco plants infected with the tobacco rattle virus posttranscriptionally silence the expression of several MEP genes, including *IspG*, *IspH*, and *IPI*, resulting in an albino phenotype (Page et al., 2004). T-DNA knockout mutant of *Arabidopsis*, *atisph*, exhibited an albino phenotype, whereas transgenic-induced gene silencing of *AtIspH* exhibited variegated albino phenotypes (Hsieh and Goodman, 2005). In the case of Z. may IspH (ZmIspH), an ethyl methanesulfonate (EMS)-induced recessive mutant and foxtail mosaic virus-induced gene silencing both showed albino phenotypes, suggesting that it is fatal to chlorophyll biosynthesis (Lu et al., 2012; Liu et al., 2016).

Interestingly, in the rice genome, the existence of two IspH alleles is predicted unlike in corn that has one *IspH* gene. Although a recent study using the CRISPR-Cas9 system presented an albino phenotype in seedlings caused by defective chloroplast biogenesis of a rice *lethal albinic seedling 1* (*las1*) mutant, corresponding to the *osisph1* (Liu et al., 2020), the rice *IspH* function has not been fully understood. In this study, we analyzed the molecular characteristics of *OsIspH1* and *OsIspH2*, performed the complementation assays of *OsIspH1* in two *hdr* mutant backgrounds, namely, *E. coli* DLYT1 strains and rice T-DNA insertional *osisph1* mutant plants, identified key residues crucial to HDR activity, and investigated the *IspH* effects on the biosynthesis of terpenoids, including chlorophylls and carotenoids, in the source and sink organs of rice plants.

## Materials and Methods

### Plant Materials and Growth Conditions

Two commercial varieties of Korean rice, *O. sativa* L. cv. Dongjin and Ilmi, were used to analyze endogenous gene expression, amplify the genes, isolate protoplasts, and perform a stable transformation. The Crop Biotech Institute in Kyung Hee University (Yongin, South Korea) generously provided a T-DNA-insertional rice mutant. Mature seeds were dehulled, surface-sterilized by 70% ethanol for 2 min and 2% sodium hypochlorite for 40 min, washed five times with distilled water, germinated for a week on Murashige and Skoog agar medium (Duchefa, Haarlem, Netherlands) in a plant growth chamber, transplanted into soil, and cultivated in a greenhouse under the conditions of 14-h light/10-h dark cycle at 28°C or in the paddy field until maturity during the summer. Mature seeds were harvested 60 days after flowering, and their endosperm color was visually compared after dehusking (TR-200 Electromotion rice husker, Kett, Tokyo, Japan) and polishing (Pearlest Polisher, Kett).

### RNA and DNA Analysis

Rice samples of diverse organs obtained at different developmental stages and 2-week-old seedlings treated with six phytohormones, including 100-μM abscisic acid (ABA), 100-μM gibberellic acid (GA3), 100-μM auxin (IAA), 100-μM kinetin (KT), 100-μM methyl jasmonic acid (MeJA), and 200-μM salicylic acid (SA), were used for total RNA extraction using mainly RNeasy Plant Mini Kit (QIAGEN, Hilden, Germany) and PureLink^®^ Plant RNA Reagent (Invitrogen, Waltham, MA, United States) for seed case. The real-time quantitative reverse transcriptase PCR (qRT-PCR) was performed using iQ™ SYBR^®^ Green Supermix (Bio-Rad) and CFX Connect™ Real-Time System (Bio-Rad), with PCR conditions and data calculated relative to the rice ubiquitin 5 gene (Os01g22490) to normalize RNA amounts, as previously described (You et al., 2020), and each qRT-PCR was performed in three technical repeats. Semiquantitative RT-PCR was performed under the following conditions: a cycle at 95°C for 3 min, 30 cycles at 95°C for 15 s, 55°C for 15 s, 72°C for 1 min/kb, followed by 3 min at 72°C with KOD FX DNA polymerase (Toyobo, Osaka, Japan).

We extracted the rice genomic DNAs using a Genomic DNA Preparation Kit (QIAGEN). The genotyping PCRs were performed using an EmeraldAmp^®^ PCR Master Mix (TaKaRa Bio, Shiga, Japan). To examine the transgene homozygosity, TaqMan PCR was performed to detect *Bar* as a transgene with an internal reference in the rice genome, i.e., the α-tubulin gene (Os11g14220), as previously described (Ha et al., 2019). Supplementary Table 10 contains all sequence information of primers.

### Cloning and Subcellular Localization Using Rice Protoplasts

The coding region of *OsIspH1* (Os03g0731900) was amplified using gene-specific primer pair from the total RNA of 10-day-old seedlings of rice (*O. sativa* L. cv. Ilmi) and cloned into the *pDONR221* vector using the Gateway^®^ BP^®^ Clonase II Enzyme Mix (Invitrogen), yielding *pDONR221-OsIspH1*. To determine the subcellular localization, *OsIspH1* was fused to the N-terminus of superfolder green fluorescent protein (sGFP) and cloned into the *pB2GW7* binary vector using Gateway^®^ LR Clonase^®^ II Enzyme Mix (Invitrogen). Protoplast isolation, DNA transfection, and confocal microscopy analysis were conducted, as previously described (You et al., 2016). The fluorescence was detected and imaged using a confocal laser scanning microscope (Carl Zeiss LSM 880, Jena, Germany).

### Site-Directed Mutagenesis and Complementation Assay in *E. coli* DLYT1 Strain

Using the *pDONR221-OsIspH1* as a template, two non-synonymous substitution mutations (H145P and K407R) in OsIspH1 were created to have one or both of each through site-directed mutagenesis with sequence-substituted primers (Supplementary Table 10). Original and mutated genes were cloned into *pMW118* (Nippon Gene, Tokyo, Japan) and transformed into the *E. coli* DLYT1 strain, which was disrupted in *LytB/EcIspH* encoding *E. coli* HDR gene (Kim et al., 2008). An *E. coli* DLTY1 strain transformed with *pTTQ18-EcIspH/LytB* was used as a positive control for complementation analysis. The complementation tests were performed on the Luria-Bertani (LB) medium containing 50 μg/ml kanamycin, 100 μg/ml spectinomycin, and 0.01% (w/v) (±)-mevalonolactone (MVA) (Sigma-Aldrich, St. Louis, MO, United States).

### Transformation and Cross-Fertilization of Rice Plants

After amplification of a double mutated gene using the *pMW118-OsIspH1^*H*145*P/K*407*R*^* as a template, a constitutive expression cassette was prepared by consecutive cloning into the *pDONR221* vector and the *p600-PGD1* vector containing a rice phosphogluconate dehydrogenase 1 promoter using the Gateway procedures (Park et al., 2010), resulting in *pIspH1^MUT^*. It was further introduced into *pGlb:stPAC* vector, in which endosperm specifically produces β-carotene (Jeong et al., 2017), resulting in *pIspH1^*MUT*^-stPAC.* Two final binary vectors were transformed *via* triparental mating of *Agrobacterium* LBA4404 harboring *pSB1* plasmids and cocultivated with embryogenic calli differentiated from matured rice seeds (*O. sativa* L. cv. Ilmi), as previously described (You et al., 2020). The putative transgenic plants were generated on a selection medium containing phosphinothricin (4 mg/L) and cefotaxime (500 mg/L) using shooting and rooting procedures, according to a previously described method (Ko et al., 2018).

*In planta* complementation assay was performed using conventional interbreeding during the flowering season at the T3 generation between heterozygous plants of *osisph1* as the female parent and homozygous *pIspH1^MUT^*-overexpressed plants as the male parent. Filial (F) 1 progenies were self-pollinated in the paddy field for three more generations to obtain homozygous seeds for transgene traits of both parents.

### Analyses of Chlorophyll and Carotenoid Metabolites

Rice leaves and dehusked seeds were ground to a fine powder using a pestle in liquid nitrogen. Total chlorophylls were extracted from rice leaf powders with 100% methanol at 70°C for 30 min using ThermoMixer Comfort (Eppendorf AG, Hamburg, Germany) at 500 rpm and centrifuged for 10 min at 4°C and 3,000 rpm. A spectrophotometer was used to measure the absorbance of the supernatant at 666 and 653 nm (Mecasys Co., Daejeon, South Korea). The chlorophyll content was calculated using the formula stated by Wellburn (1994).

Carotenoids were extracted from 100 mg powders of each rice leaf and seed, following the previous report with minor modifications (Kim et al., 2010). Notably, 0.1% ascorbic acid-containing ethanol was added to 100 mg powder to be incubated at 85°C for 5 min, after vortex-mixing for 20 s. After 10 min of saponification with potassium hydroxide (80%, w/v) in an 85°C water bath, samples were immediately placed on ice, and we added cold deionized water with β-apo-8′-carotenal (25 μg/ml) as an internal standard. Hexane-extracted carotenoid layers were separated two times by centrifugation at 1,200 × *g*. Aliquots were dried under a nitrogen stream and redissolved in 50:50 (v/v) dichloromethane/methanol solution. High-performance liquid chromatography (HPLC) was performed on a C30 YMC column (4.6 mm × 250 mm, 3 μm; Waters Corporation, Milford, MA, United States) using gradient elution at 1 ml/min with solvent A [methanol/water (92.8, v/v) with 10 mM ammonium acetate] and B (100% methyl *tert*-butyl ether) under the following conditions: 0 min, 83% A/17% B; 23 min, 70% A/30% B; 29 min, 59% A/41% B; 35 min, 30% A/70% B; 40 min, 30% A/70% B; 44 min, 83% A/17% B; 55 min, 83% A/17% B. Chromatograms were generated at 450 nm. The calibration curves were set using 5 concentrations of carotenoid standards, except for lycopene, from 0.3 to 5.0 μg/ml based on the peak area ratios obtained with the internal standard. Linearity was tested by least-squares regression analysis of the corrected peak area ratios against increasing weight ratios. The calibration curves of lutein, zeaxanthin, α-carotene, and β-carotene were linear (*r*^2^ = 0.9988–0.9998). Lycopene undergoes degradation *via* isomerization and oxidation. Thus, the quantitative calculation of lycopene was based on the peak area of 5.0 μg/ml of lycopene standard (Kim et al., 2017).

## Results

### Phylogenetic Tree Analysis of IspH Family

**FIGURE 1:**
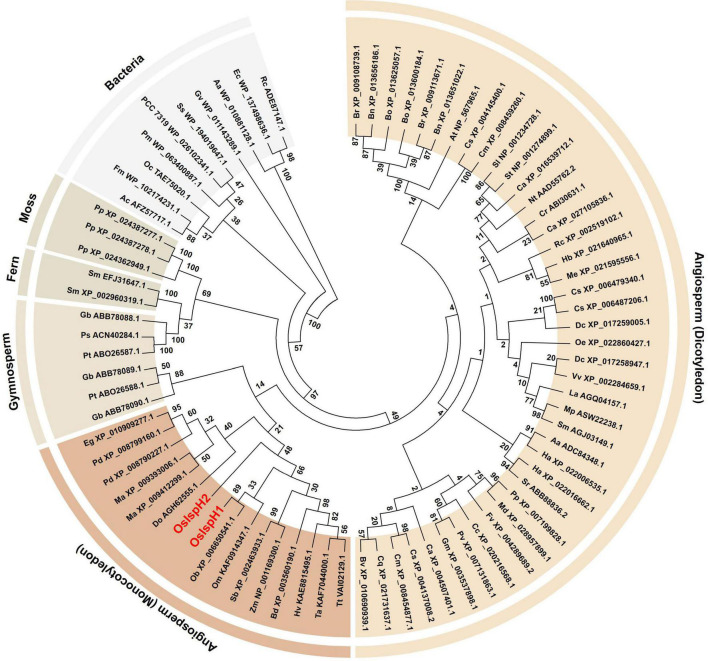
Evolutionary relationships among isoprenoid synthesis H (IspH) families. The phylogenetic tree was inferred using the maximum-likelihood method based on the JTT matrix-based model. The bootstrap consensus tree was inferred from 100 replicates. Branches corresponding to partitions reproduced in less than 50% bootstrap replicates are collapsed. The IspH family is named by a combination of a species abbreviation and an accession number, with rice IspHs marked in red letters. Evolutionary analyses were conducted on 79 amino acid sequences using MEGA7, and Supplementary Table 1 contains all information of the IspH family used in this study.

To investigate the evolutionary relationship of the rice IspH family, the predicted amino acid sequences of 79 IspHs were first collected from GreenPhylDB^1^, confirmed whether they contained a full-length open reading frame (ORF) in the National Center for Biotechnology Information Web BLAST^2^, and finally selected for the phylogenetic tree analysis using the maximum-likelihood method (Figure 1). As a result, the tree classified them into six clades, namely, bacteria, mosses, ferns, gymnosperms, monocotyledons, and dicotyledons, as they showed a sequential relationship based on general evolutionary processes. All bacteria have a single *IspH* gene, whereas plants possess one to three copies. Considering the closer relationship between cyanobacteria and plants, it was proposed that plants obtain *IspH* genes from cyanobacteria *via* endosymbiosis, which might have evolved independently with non-photosynthetic bacteria from the common prokaryotic ancestor, as previously reported (Hsieh and Hsieh, 2015). Particularly, family genes belonging to the same species were mostly grouped into the same clade or closely related, indicating that they might be multiplicated during evolutionary processes.

In the *O. sativa* genome, two *IspH* genes with 10 introns, *OsIspH1* (Os03t0731900-01) and *OsIspH2* (Os03t0732000-00), designated in this study are annotated in Rice Annotation Project Database (RAP-DB). They have 68% amino acid sequence homology and are adjacent genes about 2.8 Mbp apart in the same chromosome 3. In monocotyledon plants, *IspH* genes are analyzed to exist as two copies in date palm (*Phoenix dactylifera*), banana (*Musa acuminata*), and a domesticated-rice (*O. sativa*; AA genotype) genomes and as just a single copy in other wild rice species (*Oryza brachyantha*, FF genotype; *Oryza meyeriana*, GG genotype), sorghum, corn, barley, wheat, and so on (Figure 1). As shown in the phylogenetic tree, *IspH* genes exist mostly as two or three copies in moss, fern, and gymnosperm genomes, one or two copies in monocotyledon genomes of angiosperm plants, and mostly a single copy in bacteria, dicotyledon genomes of angiosperm plants, indicating that the copy number of *IspH* genes has evolved to increase from a single copy in bacteria to two or three copies in gymnosperms but has evolved to decrease back to a single copy in angiosperms, during the genomic evolution. Of two rice *IspH* genes, an *OsIspH1* was evolutionarily grouped with a single-copy *IspH* gene of other monocotyledon plants in a phylogenetic tree, but *OsIspH2* was distant from them (Figure 1), supposing that OsIspH1 might have a conservative IspH function in angiosperms, rather than OsIspH2 having it.

### Molecular Identification and Characterization of Rice *IspH* Genes

**FIGURE 2:**
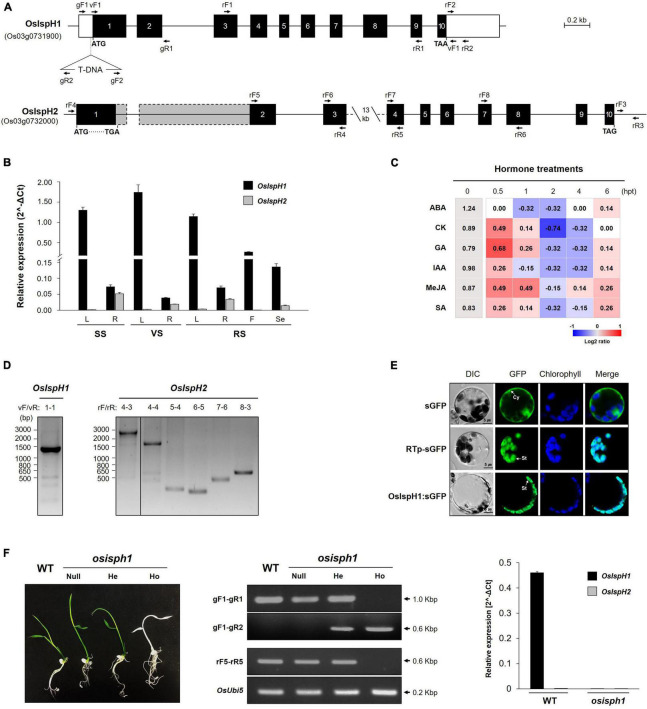
Molecular characterization of rice *IspH* genes. **(A)** Genomic structures of the *OsIspH1* and *OsIspH2* genes and verification of T-DNA position in knockout mutant *osisph1*. Solid boxes represent exon (black) and untranslated regions (white); the actual sizes of exons 1 and 2 of *OsIspH2* are represented by gray boxes with dotted line, and solid lines indicate introns. Triangle indicates the position of T-DNA insertion. **(B)** Spatial and temporal expression of *OsIspH1* and *OsIspH2* in various tissues, including leaves (L), roots (R) of the seedling stage (SS), vegetative stage (VS), and reproductive stage (RS) tissues, and florets (F) of RS and seeds (Se) harvested at 60 days after flowering. **(C)** Expression profiles of endogenous *OsIspH1* in response to different hormone treatments in 10-day-old leaves. The heatmap graph is presented by the relative log2 fold change values of each time point, which were obtained by normalizing the ΔCt values of each 0 h time point (gray-colored boxes) as individual control, and the ΔCt value of each 0 h time point is presented in column 0 hpt. **(D)** Reverse transcriptase PCRs (RT-PCRs) verify the predicted transcript sizes of *OsIspH1* (left panel) and *OsIspH2* (right panel) using rice young seedling roots. **(E)** Subcellular localization of the OsIspH1:superfolder green fluorescent protein (sGFP) fusion protein with sGFP and RTp-sGFP, which are used as cytosol (Cy)- and stroma (St)-localized markers, respectively. A confocal microscope was used to detect the sGFP fluorescence and chlorophyll autofluorescence. **(F)** Characterization of the loss-of-function mutant for *OsIspH1* using 5-day-old seedling phenotypes (left panel), genotyping and semiquantitative reverse-transcriptase PCRs (middle panel), and real-time quantitative reverse transcriptase PCR (qRT-PCR, right panel). WT, *Oryza sativa* Japonica cv. Dongjin as a wild-type control; null, nullizygous plants; He, heterozygous plants; Ho, homozygous plants. The used primer locations are indicated with arrows in **(A)** and Supplementary Table 10 shows the sequence information. All qRT-PCRs were triplicated and calculated using the ΔCt equation against the *OsUbi5* gene as an internal control. The results are expressed as the mean ± standard error (SE), and Supplementary Tables 3, 4 show the original values.

To define the structures of two rice *IspH* genes, we extracted genomic DNA sequence information from the RAP-DB^3^ and constructed their genome structures, which are equally composed of ten exons and nine introns (Figure 2A). The 1st and 3rd introns of *OsIspH2* have unusually longer sequences, 1,061 bp and 13.5 kbp, respectively, compared with 652 and 409 bp of *OsIspH1* when predicted only with the genomic DNA sequence because the cDNA of *OsIspH2* has never been reported. It meant that the functionality of *OsIspH2* was largely unknown.

To compare the expression patterns of two rice *IspH* genes, we examined them in various tissues at different developmental stages with the gene-specific primers located in a 3′-untranslated region (Figure 2B and Supplementary Table 3). The spatial expression pattern revealed that *OsIspH1* transcripts were 20–40 times higher in the leaves than roots at all developmental stages, indicating a preference for leaves. They were also expressed in florets and seeds. *OsIspH2* was not expressed in any leaf tissue and florets but was found in low levels in roots and seeds, indicating that *OsIspH1* might be mainly functional in all rice tissues. Furthermore, we examined the expression patterns of two rice *IspH* genes against the treatments of six phytohormones, namely, ABA, CK, GA, IAA, MeJA, and SA (Figure 2C and Supplementary Table 4). Transcript levels of *OsIspH1* were slightly suppressed by ABA but were induced by other five hormones in early time posttreatment, proposing different responses. Those of *OsIspH2* were barely detectable before and after treatment.

To simultaneously identify the cDNAs of two rice *IspH* genes, total RNAs were prepared from roots at the seedling stage using the spatial expression pattern in Figure 2B, and RT-PCRs were performed using diverse primer combinations (Figure 2D). *OsIspH1* exhibited a single 1,410-bp-long band (including 1,380 bp encoding 459 amino acids) as predicted in amplification, including the full-length ORFs; however, *OsIspH2* unexpectedly showed a longer single 2,705-bp band than the predictable 1,644 bp (including 1,449 bp encoding 482 amino acids). Through further RT-PCRs of *OsIspH2* with different combinations of exon-specific primers and sequencing of corresponding amplicons, we confirmed the only splicing error in the 1st intron, generating the longer 1st exon, shorter 1st intron, and quite longer 2nd exon than expected size, as indicated by the dotted line in Figure 2A. Interestingly, the extended 1st exon region contained the early stop codon, indicating that *OsIspH2* could be a non-functional gene by causing a disabled small ORF of 324 bp encoding 107 amino acids.

Accordingly, *OsIspH1* turned out to be the only functional HDR gene in rice. Its localization was examined using a GFP-fused protein transformation in rice protoplasts prepared from green seedlings and confirmed in stroma inside chloroplasts, with a similar pattern to stroma marker, rice ribulose-1,5-bisphosphate carboxylase/oxygenase small subunit transit peptide, but different from cytosol marker (Figure 2E).

To evaluate the role of *OsIspH1* in rice plants, we obtained a rice mutant with a T-DNA insertion in the region of 5′-untranslated region of *OsIspH1* (PFG_1B-11039)^4^, as depicted in Figure 2A. We confirmed that the *osisph1* knockout mutant exhibits albinism in homozygous plants based on genotyping and qRT-PCR (Figure 2F), and the albino phenotypes were observed at a 3:1 segregation ratio to identify a single-copy insertion of T-DNA, supposing that the albino phenotype might be derived from the knockout of *OsIspH1* gene functions. Similarly, another knockout mutant of an *OsIspH1* gene showed also the lethal albinism and the defectiveness of chloroplast biogenesis, which was constructed by a CRISPR-Cas9 gene-editing system (Liu et al., 2020), supposing that the albinism phenotype of *osisph1* might also be mediated by the defective chloroplast biogenesis. Taken together, *OsIspH1* is suggested to be crucial in photosynthetic pigments, including each or both chlorophylls and carotenoids, and that *OsIspH2* could not compensate for the loss of function of *OsIspH1*.

### Elucidation of Key Residues of OsIspH1 by Functional Complementation in *E. coli*

**FIGURE 3:**
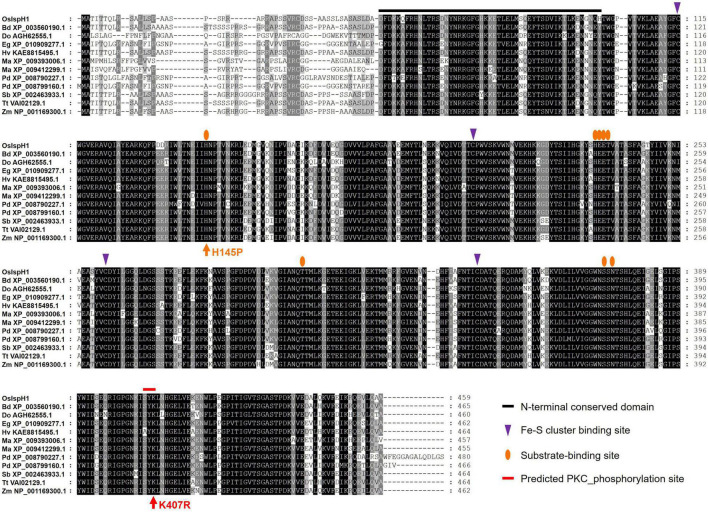
Amino acid sequence alignment among monocot plant IspHs. Supplementary Table 2 contains more information on IspH proteins.

To confirm the conservation in key residues of *OsIspH1*, we aligned amino acid sequences among 12 monocot plants, including rice (Figure 3). Previously known key residues, an N-terminal conserved domain (NCD), four Cys-residues consisting of Fe-S clusters at active sites, and eight substrate-binding sites are highly conserved among these IspHs, indicating that *OsIspH1* could function as an HDR. Interestingly, a potential key residue, such as protein kinase C (PKC) phosphorylation site in the C-terminal region, was newly predicted to be conserved among IspHs in the corresponding position of *OsIspH1*, Ser-Tyr-Lys^405––407^, through a Motif Scan analysis^5^, and Lys^407^ was also predicted as one of the ubiquitination sites through computational prediction of ubiquitination site^6^.

**FIGURE 4:**
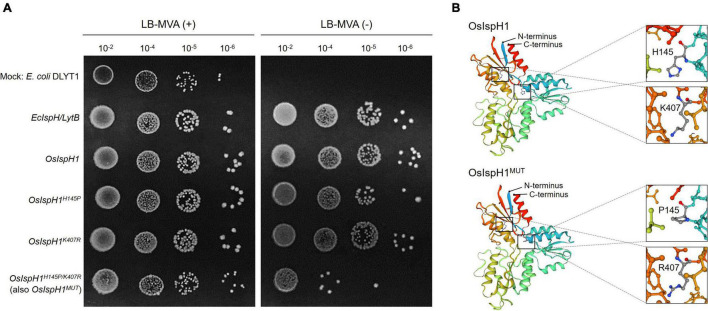
The 4-hydroxy-3-methylbut-2-enyl diphosphate reductase (HDR) enzyme assay in an *Escherichia coli* system and simulated protein structures of IspHs. **(A)** Functional complementation in *E. coli* DLYT1, an *EcIspH* lesion strain. An *E. coli IspH* gene, *EcIspH*/*LytB*, was used as a positive control. Mevalonate (MVA) (+) and (-) mean 0.01% and none of mevalonolactone, respectively. **(B)** Simulation of the 3D modeling to predict protein structure as rainbow ribbon drawing based on PDB ID: 3DNF from *Aquifex aeolicus* IspH built using SWISS-MODEL. Enlarged views showed the region with H145 and K407 residues as a ball and stick model with carbon (gray), nitrogen (blue), and oxygen (red) atoms.

Referring to the well-studied key residue of His^152^ in *Arabidopsis* (Hsieh et al., 2014), we mutated OsIspH1 to have non-synonymous substitutions in the corresponding His^145^ residue and newly predicted Lys^407^ residue into Pro^145^ and Arg^407^, respectively. Two single-mutated *OsIspH1^*H*145*P*^* and *OsIspH1^*K*407*R*^* genes and a double-mutated gene of *OsIspH1^*H*145*P/K*407*R*^* were transformed into an *E. coli IspH1/LytB*-lesion mutant strain, a DLYT1 (Kim et al., 2008). As a result, *OsIspH1*, *OsIspH1^*H*145*P*^*, and *OsIspH1^*K*407*R*^* successfully complemented the lethal phenotype of DLYT1 when *EcIspH1/LytB* was transformed as a positive control (Figure 4A). It confirmed that individual single mutation does not affect HDR activity. However, a double-substitution mutant, *OsIspH1^*H*145*P*/*K*407*R*^* (also called *OsIspH1^MUT^* hereafter), barely restored DLYT1 growth, indicating the complementary relationship of two residues for HDR enzyme activity. Additionally, to inquire whether substitution mutations of amino acids cause structural change in *OsIspH1*, we simulated the three-dimensional modeling based on PDB ID: 3DNF from *A. aeolicus* IspH using SWISS-MODEL^7^, as depicted in Figure 4B. According to a previous report, the first mutation from a positively charged His to a non-polar Pro is crucial for the “induced fit” effect to bind the substrate HMBPP (Hsieh et al., 2014). In this study, the predicted modeling showed the possibility that 1st key His^145^ residue might be faced to the Lys^407^ residue at nearly position (Figure 4B), supporting the possibility that the complementary relationship of two residues might be important for HDR enzyme activity in rice plants, although the mechanism is largely unknown.

### *In planta* Effects of OsIspH1 on the Biosynthesis of Chlorophylls and Carotenoids

**FIGURE 5:**
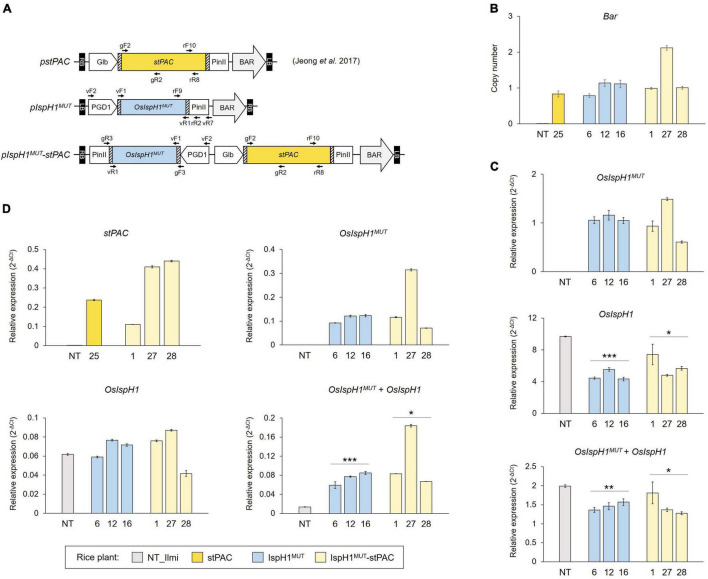
Generation of transgenic rice plants for *in planta* characterization. **(A)** Schematic representation of binary vectors for rice transformation with the indicated positions of primer sets listed in Supplementary Table 10. **(B)** TaqMan PCR to verify the transgene copy numbers using bialaphos-resistant (Bar) gene cassette. The previously reported homozygous stPAC 25 line was used as a reference for the single T-DNA insertion (Jeong et al., 2017). The qRT-PCR to examine transcript levels of transgenes, *OsIspH1^MUT^* or *stPAC*, endogenous *OsIspH1*, and combined genes, *OsIspH1^MUT^* and *OsIspH1*, in transgenic rice leaves **(C)** and seeds **(D)**, respectively. The rice ubiquitin gene (Os01g22490) was used as an internal reference for quantitative normalization, and the values were represented as the mean ± SE of three independent measurements. Statistical analyses were performed using a one-tailed Student’s *t*-test, and significant differences were indicated by a *p*-value (*p < 0.05, **p < 0.01, and ***p < 0.001).

Based on the albino phenotype of the loss-of-function mutants, *osisph1*, as depicted in Figure 2F, we constitutively overexpressed an *OsIspH1^MUT^* to elucidate how an *OsIspH1* affects chlorophyll and carotenoid biosynthesis in rice leaves and seeds. To learn more about its effects on carotenoid metabolism in seeds involved in the Golden Rice trait, the *OsIspH1^MUT^* cassette was further combined with a *stPAC* cassette, which produces β-carotene in rice endosperm (Jeong et al., 2017), as a single T-DNA vector (Figure 5A). Using three-independent homozygous T3 plants, which were selected by considering the lower copy number of one and two (Figure 5B), the expression patterns of a double-mutated transgene and an endogenous gene encoding rice HDR/IspH1 were examined in leaves (Figure 5C) and seeds (Figure 5D) of T4 rice plants. The overexpression of transgene, *OsIspH1^MUT^*, in both organs and seed-specific expression of *stPAC* was confirmed relative to non-transgenic (NT) rice plants. Interestingly, the ectopic expression of an *OsIspH1^MUT^* distinctly affected the expression patterns of endogenous *OsIspH1* in both organs. In leaves of the IspH1^*MUT*^ transgenic plants, the expression of endogenous *OsIspH1* was significantly suppressed by an average of about 50% (*P* < 0.001 in IspH1^*MUT*^ and *P* < 0.05 in IspH1^*MUT*^-stPAC), and the combined transcript levels of *OsIspH1^MUT^* and *OsIspH1* were analyzed to be also significantly lower than the normal transcript level of endogenous *OsIspH1* (*P* < 0.01 in IspH1^*MUT*^ and *P* < 0.05 in IspH1^*MUT*^-stPAC), even under the ectopic expression of *OsIspH1^MUT^* transgene (Figure 5C and Supplementary Table 5). In contrast to the suppression patterns in leaves, the combined transcript levels of *OsIspH1^MUT^* and *OsIspH1* were analyzed to be at least 4 times higher than the transcript level of endogenous *OsIspH1* (*P* < 0.001 in IspH1^*MUT*^ and *P* < 0.05 in IspH1^*MUT*^-stPAC), and the transcript levels of endogenous *OsIspH1* were not suppressed in seeds of all transgenic plant (Figure 5D and Supplementary Table 6), indicating that *OsIspH1^MUT^* was expressed four times more than the intrinsic *OsIspH1* gene. Finally, the distinct expression patterns between leaves and seeds were statistically significantly conservative in six independent transgenic plants of IspH1^*MUT*^ and IspH1^*MUT*^-stPAC, and taken collectively, it was supposed that the expression of *OsIspH1* might be differentially regulated between leaves and seeds.

**FIGURE 6:**
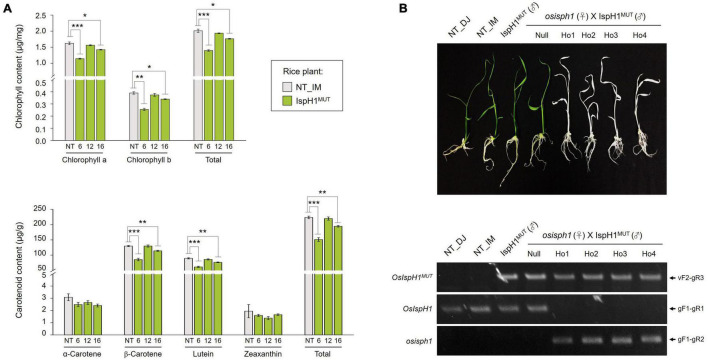
Effects of the *OsIspH1^MUT^* on rice leaves. **(A)** Contents and composition of chlorophylls and carotenoids in the leaves at T4 plants. Non-transgenic (NT) rice plants (*O. sativa* L. cv. Ilmi) and three independent IspH1^MUT^ transgenic plants were used for metabolite analysis. All data are represented as the mean ± SE of three independent measurements. Statistical analyses were performed using a one-tailed Student’s *t*-test, and significant differences were indicated by a *p*-value (*p < 0.05, **p < 0.01, and ***p < 0.001). **(B)** Functional complementation in *osisph1* rice plants. Phenotypes of 10-day-old seedlings by interbreeding between heterozygous *osisph1* and IspH1^MUT^ transgenic plants at F4 generation and genomic PCR to confirm genotypes using primer sets listed in Supplementary Table 10.

**FIGURE 7:**
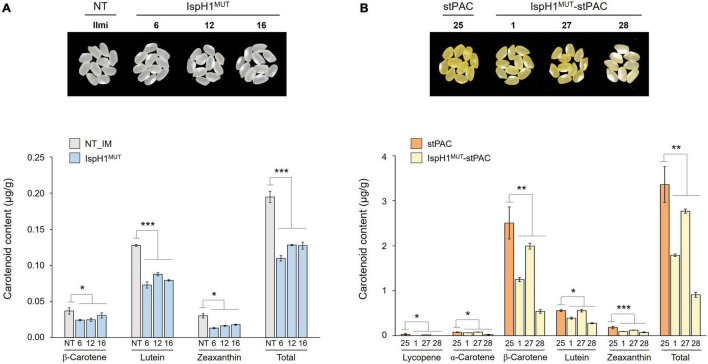
Color phenotypes and carotenoid levels in the rice seeds of IspH1^MUT^ **(A)** and IspH1^MUT^-stPAC **(B)** transgenic lines. Colors of mature seed harvested 60 days after flowering were compared, dried for 2 weeks at room temperature, threshed, dehusked, and polished in the homozygous T4 generation. Carotenoid contents were analyzed using high-performance liquid chromatography (HPLC), and all data are represented as the mean ± SE of three independent measurements. Statistical analyses were performed using a one-tailed, and significant differences were indicated by a *p*-value (*p < 0.05, **p < 0.01, and ***p < 0.001).

In the next step, we analyzed two terpene-derived photosynthetic pigments, including chlorophylls and carotenoids, using HPLC. In leaves, although there were differences between the independent transgenic plants, the total contents of chlorophylls (an average of 84.6% level, Supplementary Table 7) and carotenoids (an average of 84.3% level, Supplementary Table 8) were decreased in two transgenic plants with the significant differences compared to NT plants (Figure 6A). In addition to the functional complementation of *OsIspH1^MUT^* as HDR/IspH in *E. coli* (Figure 4A), *in planta* functional complementation was examined through cross-interbreeding between two transgenic rice plants, namely, *osisph1* and IspH1^*MUT*^. In rice plants, the albinism of *osisph1* was not restored by *OsIspH1^MUT^* overexpression at all, of which homozygosity was ascertained by genotyping (Figure 6B), indicating that OsIspH1^MUT^ was a protein defective in HDR activity in rice plants. Collectively, it was supposed that the overexpression of OsIspH1^MUT^ might cause the relative decrease in active HDR proteins through the competition of OsIspH1^MUT^ with the endogenous OsIspHs and consequently decrease chlorophyll and carotenoid contents in the leaves. In the seeds, the carotenoid content of IspH1^MUT^ (Figure 7A) and IspH1^*MUT*^-stPAC (Figure 7B) was statistically significantly reduced on the average of 61.4 and 54.2%, respectively, compared to NT, indicating a more severe pattern in seeds than leaves (Figure 7 and Supplementary Table 9). The results were considered to be closely related to the higher expression level of OsIspH1^MUT^ than the endogenous *OsIspH1* gene in the seeds.

Taken together, OsIspH1 is supposed to have crucial roles such as the rate-limiting regulation on carotenoid biosynthesis in seeds and photosynthetic pigments in leaves.

## Discussion

### OsIspH1 Is the Unique Functional HDR in Rice Plants

The MEP pathway consists of seven enzymatic steps that start with the catalysis of DXS and end with that of IspH. While the five intermediate enzymes are encoded by a single gene, DXS and IspH have been reported as one or multiple genes in diverse plants, proposing the potent roles as flux-controlling steps for terpenoid production. Plant DXSs have been classified into three clades, with different influences on the downstream pathways: DXS1s for chlorophyll biosynthesis with a housekeeping role, DXS2s for the production of specialized terpene metabolites, including artemisinins, ginkgolides, resins, and carotenoids, in different plants, and DXS3 for considerably understudied but still tentative roles in a few plants, as recently summarized (Cordoba et al., 2011; Zhang et al., 2018; de Luna-Valdez et al., 2021; Tian et al., 2021). Meanwhile, plant IspH has been reported as a single-gene family in most plant species but as a multiple-gene family of 2–3 copies in some species. In *G. biloba* and *Pinus taeda* of gymnosperm plants, GbIspH1 and PtIspH1 are for housekeeping roles, and GbIspH2 and PtIspH2 are for individually specialized ginkgolide and resin roles (Kim et al., 2008, 2009). In angiosperm plants, two *C. melo* genes showed different spatial expression: *CmIspH1* for the most abundant in all organs and *CmIspH2* in specialized contexts, such as de-etiolating seedlings and orange-fleshed fruit during ripening (Saladie et al., 2014). Thereby, it could be proposed that *IspH1*s play housekeeping roles, but *IspH2s* can contribute to plant species-specific terpene production. In the case of *O. sativa*, two *IspH* genes are predicted in the genome and have been previously presumed the different function: *OsIspH1* as a predominantly light-induced gene but *OsIspH2* as an upregulated gene in the *dxr* mutants without light response, proposing a compensating route awaiting validation (Jung et al., 2008). However, Liu et al. (2020) presented that the genome-edited *OsIspH1* defective mutant showed the albino lethal phenotype, and similarly in this study, the T-DNA mutant of *OsIspH1*, *osisph1*, showed the albino lethal phenotype (Figure 6), supposing that *OsIspH2* might not have any compensate function for it. Interestingly in this study, the sequence analysis of *OsIspH2* mRNA presented that the extended 1st exon region had an early stop codon to consequentially generate the truncated-short polypeptide fragments, which was spliced at the quite different region from the predicted-splicing position provided by the RAP-DB (Figures 2A,D). Collectively, an *OsIspH2* gene is supposed to be non-functional, indicating that an *OsIspH1* is a unique functional HDR gene in rice.

### The Complementary Relationship of His^145^ and Lys^407^ Residues Is Crucial for the HDR Activity of OsIspH1 in Both Bacteria and Rice Plants

The X-ray crystal structure of IspH protein has been first reported in the hyperthermophilic eubacterium *A. aeolicus* (Rekittke et al., 2008), its critical residues have been elucidated mainly in bacterial systems, such as *A. aeolicus*, *E. coli*, and cyanobacteria (Rekittke et al., 2008; Grawert et al., 2009; Hsieh and Hsieh, 2015). Meanwhile, the plant-type IspH structure has also been reported in *Arabidopsis* and the same key residues to bacterial ones by complementation assay in *E. coli isph* mutant (Hsieh et al., 2014). By Hsieh et al. (2014), two bacterial critical residues showed the difference in the corresponding ones of *Arabidopsis*, His^152^ and His^241^, not individually but cooperatively critical for HDR activity with more significance of His^152^ than His^241^. In this study, we found another conserved motif, referred to as a predicted PKC phosphorylation site in Figure 3, and to determine whether it could be a novel crucial residue of OsIspH1, we mutated each or together at both positions of His^145^ and Lys^407^ to generate *OsIspH1^*H*145*P*^* and *OsIspH1^*K*407*R*^*, and *OsIspH1^*H*145*P*/*K*407*R*^*, and the former is the corresponding site to His^152^ of AtIspH, and the latter is a newly predicted site for PKC phosphorylation or ubiquitination in this study. The complementary experiments in the *isph* mutants of bacteria and rice plants showed that the simultaneous mutation of His^145^ and Lys^407^, an OsIspH1^*H*145*P*/*K*407*R*^, largely reduced the HDR activity of OsIspH1 not only in the bacteria expression system (Figure 4A) but also in the rice plant system (Figure 6B), but every single mutation did not have any effects on the HDR activity of it in bacteria system. These results supposed that the complementary relationship of two residues, namely, His^145^ and Lys^407^, might be crucial to the HDR activity of OsIspH1.

### OsIspH1 Plays an Important Role in the Biosynthesis of Chlorophylls and Carotenoids, Through the Differential Expression Patterns Between Leaves and Seeds

The regulation of plant genes involved in MEP and MVA pathways occurs at multiple levels of transcription, posttranscription, translation, and posttranslation (Cordoba et al., 2009; Hemmerlin, 2013; Vranova et al., 2013). Particularly, plant IspHs contribute to the MEP pathway primarily through transcriptional regulation with tissue specificity and different responses against environmental conditions, such as light, circadian, mechanical wounding, and fungal elicitors in diverse plants (Kim et al., 2008, 2009; Cordoba et al., 2009; Ma et al., 2017). To scrutinize the intrinsic rice IspH regulation, we constitutively overexpressed the malfunctional OsIspH1^MUT^ (Figure 5) in rice plants, which was identified in two *hdr* mutants, namely, *E. coli* DLYT1 strains (Figure 4) and rice *osisph1* plants (Figure 6B). Interestingly, the effects of *OsIspH1^MUT^*-ectopic expression were observed to be quite differential between rice leaves and seeds. In leaves, not only the transcript level of an endogenous *OsIspH1* was downregulated, but also the combined transcript level of endogenous *OsIspH1* and exogenous *OsIspH1^MUT^* remained lower than that of NT (Figure 5C). In contrast, the expression of an endogenous *OsIspH1* was not downregulated in the seeds and remained higher than that of NT (Figure 5D), suggesting that the expression of an intrinsic *OsIspH1* gene was differentially regulated in leaves and seeds. Similarly, organ-specific differential roles of OsDXS2 and OsDXR on carotenoid accumulation between leaves and seeds have also been reported (You et al., 2020). Collectively, these results suggest that the DXS, DXR, and IspH steps belonging to the MEP pathway might be regulated to differentially affected carotenoid accumulation between leaves and seeds.

Despite such differential regulation, the ectopic expression of *OsIspH1^MUT^* simultaneously caused the decrease of chlorophyll and carotenoid content in leaves and seeds (Figures 6, 7), and the reduction patterns were proportional to the overexpression patterns of *OsIspH1^MUT^*, indicating the relative decrease in active HDR enzyme function by competition between endogenous OsIspH1 and OsIspH1^MUT^. In other words, the intrinsic OsIspH1 is supposed to play an important role in the biosynthesis of chlorophyll and carotenoid levels in rice leaves and seeds and to be a promising target to biofortify the functional terpene metabolites in rice plants.

## Data Availability Statement

The original contributions presented in the study are included in the article/Supplementary Material, further inquiries can be directed to the corresponding authors.

## Author Contributions

S-HH coordinated the project, supervised the manuscript, and was responsible for all contacts and correspondence. MY contributed to the interpretation of data for the work, and revised it critically for important intellectual content. YL performed all the experiments *in planta* and wrote the draft of the manuscript. JK and S-AB analyzed the contents of chlorophylls and carotenoids. J-SY contributed to the experiments in *E. coli.* All authors have read and approved the final manuscript.

## Conflict of Interest

The authors declare that the research was conducted in the absence of any commercial or financial relationships that could be construed as a potential conflict of interest.

## Publisher’s Note

All claims expressed in this article are solely those of the authors and do not necessarily represent those of their affiliated organizations, or those of the publisher, the editors and the reviewers. Any product that may be evaluated in this article, or claim that may be made by its manufacturer, is not guaranteed or endorsed by the publisher.

## Footnotes

1
https://www.greenphyl.org/cgi-bin/index.cgi

2
https://blast.ncbi.nlm.nih.gov/Blast.cgi

3
https://rapdb.dna.affrc.go.jp/

4
http://signal.salk.edu/cgi-bin/RiceGE

5
http://myhits.isb-sib.ch/cgi-bin/motif_scan

6
http://bdmpub.biocuckoo.org/prediction.php

7
https://swissmodel.expasy.org/
